# Differences in Resilience, Psychological Well-Being and Coping Strategies between HIV Patients and Diabetics

**DOI:** 10.3390/healthcare10020266

**Published:** 2022-01-29

**Authors:** Cristina Rivera-Picón, María Hinojal Benavente-Cuesta, María Paz Quevedo-Aguado, Pedro Manuel Rodríguez-Muñoz

**Affiliations:** 1 Faculty of Health Sciences, Nursing, Pontifical University of Salamanca, 37002 Salamanca, Spain; mhbenaventecu@upsa.es (M.H.B.-C.); mpquevedoag@upsa.es (M.P.Q.-A.); 2 Faculty of Nursing and Physiotherapy, University of Salamanca, 37008 Salamanca, Spain; pedromrodriguez@usal.es; 3 Departament of Nursing, Maimonides Biomedical Research Institute of Cordoba, 14005 Cordoba, Spain

**Keywords:** diabetes mellitus, HIV, resilience, psychological well-being, coping strategies

## Abstract

The aim of the study was to determine the differences in resilience, psychological well-being and coping strategies between patients with HIV and diabetics. The sample included a total of 400 subjects (199 patients with HIV and 201 subjects with diabetes). The instruments applied for data collection were a sociodemographic data questionnaire, the Resilience Scale (Wagnild and Young), the Ryff Psychological Well-being Scale and the Coping Strategies Questionnaire (Sandín and Chorot). The data collection period was approximately 2 years (between February 2018 and January 2020). Based on the results of our work it was found that the subjects with HIV had lower scores than the diabetic subjects in all the resilience factors, except for the factor “feeling good alone”. In addition, the subjects with HIV scored significantly lower than the diabetic subjects on all the variables of psychological well-being. Subjects with HIV used problem-solving coping, social support seeking, positive reappraisal, religious coping and avoidance coping with less frequency than diabetic subjects. However, they used more negative auto-focused coping compared to diabetic subjects. Therefore, subjects with HIV show a different psychological pattern in relation to resilience, psychological well-being and use of coping strategies compared to diabetic subjects.

## 1. Introduction

Currently, due to factors such as the development of medical technology, scientific advances, new lifestyles and an ageing population, the presence of chronic diseases in society is growing rapidly [1]. The increase in these pathologies is of great importance because the diagnosis of a chronic disease can be a profound, impactful experience. This is associated with the fact that chronicity has complex physical, psychological and social implications, requiring adaptation to new lifestyles, which requires effort and improvement [2,3].

For this reason, certain psychological variables such as resilience, psychological well-being and coping strategies take on great relevance in the approach to chronic diseases. These psychological constructs are related to the prevention and evolution of these pathologies. Resilience is considered to be the ability of the person to deal with the disease, allowing some control over the negative impact of the consequences derived from it [4,5,6,7,8]. The type of coping strategies that subjects use to adapt to their disease condition can anticipate the impact caused by said pathology on the person, since certain strategies can mediate and cushion the effects of stress. Therefore, different authors contend that an active coping style is associated with a better quality of life and greater psychological well-being [9,10]. Psychological well-being has implications for the health of the subjects, as it intervenes in the recovery of illnesses and in the maintenance of health [11].

However, each chronic disease has different processes and characteristics. Therefore, this study analyzed the differences in resilience, psychological well-being and coping strategies in two diseases with great differences in their characteristics: diabetes mellitus and HIV infection.

Lifestyle modification is essential in the treatment of diabetes mellitus. Therefore, this pathology requires a high capacity for adaptation and the modification of habits. All this, and the complications that the disease can induce, have an impact on the lives of diabetic patients, a term used for stylistic convenience [12,13,14]. On the other hand, HIV infection is a highly complex pathology, in which the subject has to face numerous physiological, sociocultural, economic and psychological stressors. For this reason, patients with HIV, in addition to the changes in lifestyle that the diagnosis requires, face social stigma, myths and negative beliefs associated with this disease. Moreover, in many cases, they suffer greater discrimination and less social support [15,16,17]. In this way, the psychological impact of HIV infection on the subjects who suffer from it is highlighted, due to the difficulties and social stigma that this entails [18,19]. These characteristics show a clear difference between both pathologies, with more difficulties and less acceptance for HIV patients. For this reason, although both chronic diseases have been stigmatized as diseases of the patient’s fault, the stigma of HIV is more pronounced. This stigma dates back to the beginning of the HIV epidemic, when it was called the 4 “H” disease, since the first cases occurred in homosexual men, heroin users, patients from Haiti, and hemophiliacs [20]. Currently, the area of HIV Surveillance and Risk Behaviors, in 2017, reported that the most frequent transmission (54.3%) occurs in men who have sex with men, followed by heterosexual transmission, which represents 28.2 two%. The remaining 3.1% corresponds to transmission associated with parenteral drug use. Thus, due to the origin and main transmission mechanisms of HIV, the stigma associated with this disease is greater than that of diabetes.

Psychological variables can contribute to the control and development of chronic disease. Therefore, it is important that health care strategies for these patients include both clinical and psychological elements, with the aim of promoting health, greater well-being and better quality of life. Thus, due to the impact at all levels of chronic diseases on people, we justify that the subject of study is fundamental at present. Knowing the diseases that are related to a worse adaptation or a worse level of psychological well-being is important to be able to focus on effective and individualized health interventions. Therefore, the objective of this study was to analyze the differences in resilience, psychological well-being and coping strategies between subjects with HIV and diabetics.

## 2. Materials and Methods

### 2.1. Aim and Design of the Study

To analyze the differences in resilience, psychological well-being and coping strategies between subjects with HIV and diabetics. The study had a non-experimental cross-sectional design with a correlational objective.

### 2.2. Participants

The total sample (*N* = 400) consisted of subjects with a diagnosis of diabetes or HIV infection. First, the sample of subjects diagnosed with HIV (*N* = 199) was collected at the Salamanca University Assistance Complex, specifically, at the Salamanca University Hospital. These patients voluntarily participated in the study after attending their scheduled appointment in the nursing consultation in the infectious diseases unit. Therefore, this subsample was obtained via incidental sampling, and it is representative since the population of subjects with HIV in the Hospital Clínico de Salamanca is approximately 600 patients. The University Hospital of Salamanca (HUS) is framed in the Public Health Service of the Autonomous Community of Castilla y León (SACYL) and is configured as a benchmark of excellence for the provision of specialized health care and for the development of the research function. Salamanca is even at the forefront of research and innovation in infectious diseases.

After obtaining the sample of HIV subjects, the sample of diabetic patients (*n* = 201) was selected, following sampling by quota with ranges of age, sex and equivalent educational level, with the aim of obtaining homogeneous subsamples. For the selection of the subsample made up of diabetic patients, we covered different hospital areas. The collection was performed from the Diabetological Unit of the Hospital Clínico De Salamanca and the Internal Medicine hospitalization floors of the same hospital.

In both subsamples, to participate in the project, the following inclusion criteria had to be met: subjects had to be of legal age, voluntarily participate in the study, and have a confirmed diagnosis of HIV or diabetes. Exclusion criterion included, suffering from any medical or psychological disease or disorder that prevented the patient from completing the study or signing the informed consent.

### 2.3. Data Collection

The data collection period was approximately 2 years (between February 2018 and January 2020). The data were collected through different questionnaires, detailed below:

#### 2.3.1. Sociodemographic Data Questionnaire

Information related to sociodemographic variables was collected through an instrument consisting of a series of sociodemographic questions and information related to health. The variables collected through this questionnaire were:

Health status (subjects with HIV or subjects with diabetes)

Sociodemographic variables studied (sex, educational level, age and marital status). It should be noted that the age variable was originally recorded quantitatively and was later categorized into subjects aged 43 years or younger, 44 to 50 years old, 51 to 55 years old, and people 56 years old or older. It was carried out in order to obtain equivalent and homogeneous samples. By making more general classifications, it was easier to obtain diabetic subjects with the desired characteristics.

#### 2.3.2. Resilience Scale (Wagnild and Young)

The Resilience Scale was created by Wagnild and Young in 1993 and adapted into Spanish by Novella (2002). This instrument is made up of 25 items, with a response range from 1, totally disagree, to 7, totally agree. The participants indicate the degree of agreement with the item, since all are written directly.

This scale in turn measures five resilience factors: (1) personal satisfaction, (2) equanimity, (3) feeling good alone, (4) self-confidence and (5) perseverance. The overall internal consistency measured through the Cronbach α coefficient had a value of 0.88.

#### 2.3.3. Ryff Psychological Well-Being Scale

The Psychological Well-being Scale was developed by Ryff in 1989 and versioned by Van Dierendonck in 2004. The adaptation to Spanish of this scale was carried out by Díaz et al. (2006) [21]. The scale was developed to measure psychological well-being following the Ryff model. This author proposed a multidimensional model of psychological well-being made up of six dimensions: (1) self-acceptance, (2) positive relations, (3) autonomy, (4) environmental mastery, (5) purpose in life and (6) personal growth.

Van Dierendonck’s version consists of a 39-item test with 6 response options (from 1, totally disagree to 6, totally agree). The version proposed by Díaz and collaborators, used in the present study, has 29 items.

#### 2.3.4. Coping Strategies Questionnaire (Sandín and Chorot)

This scale was developed by Sandín and Chorot in 2002. The Coping Strategies Questionnaire is a shortened and revised version of a previous version created in 1999 by Sandín, Valiente and Chorot, the Scale of Coping Strategies—Revised (EEC-R). The scale allows the study of seven dimensions of coping: (1) social support seeking, (2) overt emotional expression, (3) religious coping, (4) problem-solving coping, (5) avoidance coping, (6) negative auto-focused coping and (7) positive reappraisal. A second order analysis was performed with the existence of two more general dimensions. The first of them, rational coping, included problem-solving coping, positive reappraisal and social support seeking. The other dimension, emotional coping, included negative auto-focused coping and overt emotional expression. The scale is made up of 42 items with a response range that goes from 0, never, to 4, almost always. Each coping factor/dimension includes seven items, with the total variance explained by the seven factors being 55.3% [22].

### 2.4. Ethical Considerations

The study was authorized by the University Care Complex of Salamanca and by the Primary Care Management of Salamanca. It also received a favorable report from the Research with Medicines in the Health Area Ethics Committee of Salamanca, with CEIC code PI02/01/2018. In addition, the participation of the subjects was voluntary, ensuring the confidentiality and anonymity of the participants, who were provided with an information sheet and were asked to complete the informed consent form.

### 2.5. Data Analysis

Data analysis was carried out using the statistical program International Business Machines (IBM) Statistics Package for the Social Sciences (SPSS) version 25 (IBM Corp., Armonk, NY, USA).

A descriptive analysis of the sociodemographic variables was carried out in terms of sample size and percentages. The analysis of the differences between the subsamples is presented. Due to the nature of the sociodemographic variables (categorical), Pearson’s χ^2^ test was used, using Cramer’s V to determine the effect size. To interpret the magnitude of the effect found, the values proposed by Cohen (1988) were used: 0.10, 0.30 and 0.50, which are interpreted as small, moderate and large, respectively.

Multivariate analysis of variance (MANOVA) was used to determine the differences in resilience, psychological well-being and coping strategies between HIV-positive participants and diabetic patients. Before applying the MANOVA, the assumptions corresponding to this technique were verified. The assumptions to satisfy with this technique are three. The assumption of independence is ensured with the proven one. In the case of the assumption of normality, the Kolmogorov–Smirnov test was obtained, taking into account that the MANOVA is robust to noncompliance with this assumption with samples greater than 100 subjects [23]. The last assumption is that of homoscedasticity. To evaluate this assumption, the Box test was obtained. In this case, given the noncompliance, the use of robust contrast statistics is recommended, such as Pillai’s Trace and Dunnet’s C [23,24]. Therefore, these tests were chosen for the interpretation of the results.

The level of statistical significance used throughout the study was 0.05 with a 95% confidence interval.

## 3. Results

### 3.1. Descriptive Analysis

Table 1 shows the main descriptive results regarding sociodemographic variables. The total sample consisted of 400 subjects. Most of it was made up of males (*N* = 294). In relation to marital status, we observed that the total sample was made up of the same number of married subjects/couples as single, widowed or belonging to another group (*N* = 181). Most of the subjects had an educational level equivalent to secondary school or lower (*N* = 328) and with an age range between 44 and 50 years (*N* = 124).

### 3.2. Variables Related to Health Status

Table 2 shows the descriptions of the sociodemographic variables based on the health status of the participants. Based on the health status of the patients, two categories were distinguished: HIV patients (*N* = 199) and diabetics (*N* = 201). Approximately, each group consisted of 50% of the total sample. No significant differences were obtained regarding sex, educational level and age (*p* > 0.05). The only significant variable was marital status ($\chi^{2}=42.484;\;p<0.01)$. However, when interpreting Cramer’s V value, we observed that V = 0.322, so we can conclude that the effect is moderate.

Resilience, psychological well-being and coping strategies: differences between subjects with HIV and diabetes mellitus

The MANOVA technique was applied to determine the differences in resilience, psychological well-being and coping strategies, between subjects with HIV and diabetic patients.

Table 3 shows the results of the multivariate test. From the Pillai trace, it was observed that there were significant differences between groups (F(df) = 13.851(18); *p* < 0.001) The power was high (1.000) and the effect size was large ($\eta_{p}{}^{2}$ = 0.397).

Significant differences were detected (Table 4) in the resilience factors, except in resilience: feeling good alone, (F(gl) = 3.490 (1); *p* = 0.062). The power was moderate to low (0.462) and the effect size was small ($\eta_{p}{}^{2}$ = 0.009).

Significant differences were found in all dimensions of psychological well-being. Significant differences were detected in all the coping strategies studied, except in the overt emotional expression strategy. The power was low (0.308) and the effect size was small ($\eta_{p}{}^{2}$ = 0.005).

Table 5 shows the differences, according to the resilience factors, between groups using Dunnett’s C test. There were no differences between the two groups in the resilience variable: feeling good alone (HIV-diabetes = −0.45, ES = 0.238, CI95% = (−1.01, 0.12)). The subjects with HIV obtained significantly lower scores than the diabetic subjects for the rest of the variables.

Table 6 shows the results of the variables related to psychological well-being. HIV-positive subjects scored significantly lower than diabetic subjects on all well-being variables. In the variable positive relations, one of which has a higher effect size, the subjects with HIV scored almost five points less than the subjects with diabetes (HIV-diabetes = −4.78, ES = 0.484, CI = [−5.92–3.64]).

Table 7 shows the results in relation to the coping strategies. HIV-positive subjects used problem-solving coping and social-support seeking strategies less frequently than diabetic subjects. However, they used more negative auto-focused coping compared to diabetic subjects (HIV-diabetes = 2.45, ES = 0.402, CI = (1.50, 3.39)).

Regarding the variable social support seeking, which had the highest effect size, the subjects with HIV scored almost seven points less than the subjects with diabetes (HIV-diabetes = −6.81, ES = 0.689, CI = (−8.43, −5.18)).

## 4. Discussion

In most published studies, the impact of resilience, coping strategies and psychological well-being in different chronic diseases has been addressed [25,26,27,28]. In this way, it is known that the highest levels of resilience and psychological well-being are related to improvements in mental and physical health, reducing the risk of depression, disability and poor quality of life, among other benefits. [4,5,29]. Moreover, certain coping strategies are considered possible health protective factors and promoters of psychological well-being [30].

Therefore, due to the benefits of these psychological resources on the health of subjects, we consider it important to know what differences exist in resilience, psychological well-being and in the coping strategies used in different health states, in order to create personalized interventions taking into account the aspects that are generally most affected according to the pathology.

Based on the results of our work, it was found that the subjects with HIV had significantly lower scores than the diabetic subjects in all the resilience factors, except for the feeling-good-alone factor. A study by Gheshlagh et al. (2016) showed that individuals with different chronic diseases had a lower level of resilience than healthy subjects [28]. However, our results show that it is necessary to develop more research to evaluate and compare resilience in specific chronic diseases, which will determine the differences in resilience depending on the pathology. Due to the fact that the presence of such investigations is very scarce, few studies have compared the level of resilience in subjects with HIV and diabetic patients, although there have been some investigations with similar objectives. Research by Willrich et al. (2016), which compared the resilience level of diabetic subjects and subjects with chronic kidney disease, found that diabetes patients had a higher resilience level than kidney patients [31]. These results are not directly comparable with those of our study. However, they also reflect that diabetic subjects scored higher in most of the resilience factors.

Regarding the dimensions of psychological well-being, the subjects with HIV scored less than the diabetics in all the dimensions of this construct. It has been stated that the stigma associated with HIV is often associated with psychological problems in these patients [17,32]. This could explain why, in the present work, the subsample of patients with HIV had lower levels of psychological well-being and resilience. However, in relation to the dimensions of psychological well-being, there are not enough studies with which we can compare our results.

Additionally, based on coping strategies, our results show that subjects with HIV used strategies for problem-solving coping, social-support seeking, positive reappraisal, religious coping and avoidance coping less frequently than diabetic subjects. In contrast, HIV patients used more negative auto-focused coping. Referring to the general classification of coping strategies, we can highlight that HIV patients employ fewer rational strategies than subjects with diabetes. However, HIV patients scored higher on emotional strategies, specifically on negative auto-focused coping, than did individuals with diabetes.

All this is important if we remember that rational coping, characterized by the mobilization of the patient to deal with the disease, is associated with a good adaptation to the disease and an adequate degree of well-being [30,33]. Against this, well-being is negatively related to strategies such as negative auto-focused coping. This is because the lack of coping is linked to a lack of resources to face difficulties, such as self-blame for a situation [34,35].

However, in the current bibliography there is not enough evidence on the differences in the use of coping strategies in subjects with chronic disease. Furthermore, studies that have analyzed coping strategies in patients with HIV present contradictory results, which reflects the need for further research in this area. Thus, Sun et al. (2007) stated that, in subjects with a diagnosis of HIV, confrontation was the most-used coping style [36]. Other studies identified problem-solving coping, social-support seeking and avoidance coping as frequently used coping strategies [37,38]. All these results differ from those found in our work, in which the strategies based on the problem-solving coping and social-support seeking and avoidance were less used by the subjects with HIV compared to the diabetic subjects. In our study, social-support seeking was the least used by the subjects with HIV compared to the other group. In line with this result, some studies have shown that a feeling of shame and fear can prevent these patients from disclosing the experiences of their illness and emotional distress to family or friends; they use this type of coping strategy less frequently [39,40,41]. Our study found that subjects with diabetes scored higher on social-support seeking strategies compared to those with HIV. This coincides with the results of other studies, such as those of Rondón and Lugli (2013) and Ledon et al. (2007), who showed that social-support seeking was one of the strategies most frequently used by diabetics to face their disease [42,43]. On the other hand, the research by Iglesias-Rey et al. (2013) found that religious coping was frequently used to deal with HIV [44]. However, Burns et al. (2016) stated that the moral connotations associated with HIV infection can turn the religious community into a stigmatizing atmosphere in such patients, which can lead them to withdraw from said community [45]. Our results support this last hypothesis, since subjects with HIV used religious coping less frequently than subjects with diabetes as a strategy to cope with the disease.

In relation to the strategies used by diabetic subjects, different studies that analyzed this variable according to the general classification coincide in considering that problem-solving coping was the most-used strategy by these subjects [46,47]. These results are consistent with those of our study, which reflect that diabetic subjects used rational coping strategies (problem-solving coping, positive reappraisal and social-support seeking) more frequently than HIV-positive subjects. On the other hand, the research by Rondón and Lugli (2013) shows that social support seeking was the strategy most frequently used by diabetics to face their disease [42]. These results are also consistent with those reflected in our study, since diabetic subjects obtained seven more points in the social support strategy than subjects with HIV.

In this way, patients with HIV could be using emotional strategies to control the disease, inadequate for the optimal balance of health. This can be explained by the fact that HIV disease has a considerable emotional impact on the individual [15]. For this reason, we interpret that these patients do not feel they have sufficient capacity to actively face the adverse situations derived from the disease, thus employing, for the most part, strategies based on negative auto-focused coping. In addition, this greater use of emotional strategies could explain the lower levels of resilience and psychological well-being in this group of patients. However, diabetic subjects used more rational strategies, which may also explain the previous results, and showed greater resilience and psychological well-being in these subjects compared to HIV patients. Thus, diabetic subjects could benefit from the positive effects of rational strategies.

Among the limitations of the present study, the non-differentiation between the type of diabetes in this subsample stands out, which has been controlled as a confounding factor in other studies [48,49,50,51]. Future research should try to replicate the results obtained here by taking into account the type of diabetes diagnosed. Another limitation of the present study is the type of sample: examined not occasionally. This diminishes the external validity of the study, making it difficult to generalize the results. However, given the characteristics of the sample we have accessed, it is not very functional to carry out random sampling.

## 5. Conclusions

In conclusion, subjects with HIV show a differentiated psychological pattern in relation to resilience, psychological well-being and the use of coping strategies compared to diabetic subjects.

In this way, the results of this study allow us to predict and anticipate those subjects with a higher risk of using maladaptive coping strategies and who may experience lower levels of resilience and psychological well-being depending on the chronic disease they suffer from. For this reason, the results found here are of special interest in the creation of secondary prevention plans, focusing on the psychological aspects most frequently affected in each specific disease. However, to continue developing prevention strategies, it is necessary to expand research with more chronic diseases in order to make adaptations to each disease. The scope of our research determines that the proposal presented only includes two chronic pathologies: HIV and diabetes mellitus. Likewise, as a future line, the development of qualitative research is proposed to carry out a comparative examination of coping processes.

## Figures and Tables

**Table 1:** Descriptive: sociodemographic variables.

	*N*	%
Sex		
Woman	106	26.5%
Man	294	73.5%
Marital status		
Married/couple	181	45.3%
Single/widowed/others	181	45.3%
Separated/divorced	38	9.5%
Level of studies		
Secondary or lower	328	82.0%
Superior	72	18.0%
Age		
43 years or younger	96	24.0%
44 to 50 years	124	31.0%
From 51 to 55 years old	101	25.3%
56 years or older	79	19.8%

*N*: number of subjects; %: percentage.

**Table 2:** Sociodemographic variables based on health status.

	HIV		Diabetes		Total		Ji	TE	*p*
	*N*	%	*N*	%	*N*	%			
N° participants	199	49.8%	201	50.2%	400	100%			
Sex									
Woman	48	24.1%	58	28.9%	106	26.5%	1.151	0.054	0.283
Man	151	75.9%	143	71.1%	294	73.5%			
Marital status									
Married/couple	58	29.1%	123	61.2%	181	45.3%	42.484	0.322	0.000
Single/widowed/other	117	58.8%	64	31.8%	181	43.3%			
Separated/divorced	24	12.1%	14	7.0%	38	9.5%			
Level of studies									
Secondary or lower	168	84.4%	160	76.9%	328	82%	1.574	0.063	0.210
Superior	31	15.6%	41	20.4%	72	18%			
Age									
43 years or younger	50	25.1%	46	22.9%	96	24%	5.521	0.117	0.137
44 to 50 years	61	30.7%	63	31.3%	124	31%			
From 51 to 55 years old	57	28,6%	44	21.9%	101	25.3%			
56 years or older	31	15,6%	48	23.9%	79	19.8%			

*N*: Number of subjects; %: percentage; $\chi^{2}:\;chi-squared;$ TE: effect size.

**Table 3:** Multivariate tests: Pillai trace.

Effect	Value	F	Hypothesis df	Error df	*p*	$\eta_{p}{}^{2}\;$	Observed Power
Intersección	0.983	1241.099	18	378	<0.001	0.983	1.000
Health status	0.397	13.851	18	378	<0.001	0.397	1.000

$\eta_{p}{}^{2}:$ partial eta squared; df: degrees of freedom.

**Table 4:** Effects tests between subjects: health status.

Dependent Variable	F	Sig.	$\eta_{p}{}^{2}$	df	Observed Power
RS Personal satisfaction	8.980	0.003	0.022	1	0.848
RS Equanimity	21.408	<0.001	0.051	1	0.996
RS Feeling good alone	3.490	0.062	0.009	1	0.462
RS Perseverance	15.129	<0.001	0.037	1	0.973
RS Self-confidence	13.785	<0.001	0.034	1	0.959
PWS Self-acceptance	41,579	<0.001	0.095	1	1.000
PWS Autonomy	6.027	0.015	0.015	1	0.688
PWS Purpose in life	71.459	<0.001	0.153	1	1.000
PWS Positive relations	97.419	<0.001	0.198	1	1.000
PWS Environmental mastery	51.459	<0.001	0.115	1	1.000
PWS Personal growth	18.229	<0.001	0.044	1	0.989
CS Problem-solving coping	18.918	<0.001	0.046	1	0.991
CS Social support seeking	97.650	<0.001	0.198	1	1.000
CS Positive reappraisal	12.591	<0.001	0.031	1	0.943
CS Negative auto-focused coping	36.971	<0.001	0.086	1	1.000
CS Overt emotional expression	2137	0.145	0.005	1	0.308
CS Religious coping	26.354	<0.001	0.063	1	0.999
CS Avoidance coping	18.073	<0.001	0.044	1	0.989

$\eta_{p}{}^{2}$ : partial eta squared; RS: resilience; PWS: psychological well-being; CS: coping strategies.

**Table 5:** Multiple comparisons: resilience factors.

Dependent Variable	(I) Subject Health Status	(J) Subject Health Status	Mean Difference (I-J)	Std. Error	CI 95%	
					Lower Bound	Upper Bound
RS personal satisfacción	VIH	Diabetes	−1.03 *	0.343	−1.84	−0.22
RS Equanimity	VIH	Diabetes	−1.33 *	0.287	−2.01	−0.65
RS Feeling good alone	VIH	Diabetes	−0.45	0.238	−1.01	0.12
RS Perseverance	VIH	Diabetes	−2.26 *	0.580	−3.63	−0.89
RS Self-confidence	VIH	Diabetes	−2.34 *	0.629	−3.82	−0.85

The Dunnett C test was used. * significant differences; RS: resilience; Std. error: standard error; CI: confidence interval.

**Table 6:** Multiple comparisons: dimensions of psychological well-being.

Dependent Variable	(I) Subject Health Status	(J) Subject Health Status	Mean Difference (I-J)	Std. Error	CI 95%	
					Lower Bound	Upper Bound
PWS Self-acceptance	VIH	Diabetes	−2.38 *	0.370	−3.26	−1.51
PWS Autonomy	VIH	Diabetes	−1.35 *	0.548	−2.64	−0.05
PWS Purpose in life	VIH	Diabetes	−3.92 *	0.464	−5.02	−2.83
PWS Positive relations	VIH	Diabetes	−4.78 *	0.484	−5.92	−3.64
PWS Environmental mastery	VIH	Diabetes	−2.90 *	0.404	−3.85	−1.95
PWS Personal growth	VIH	Diabetes	−1.45 *	0.338	−2.24	−0.65

The Dunnett C test was used. * significant differences; PWS: psychological well-being; Std. error: standard error; CI: confidence interval.

**Table 7:** Multiple comparisons: coping strategies.

Dependent Variable	(I) Subject Health Status	(J) Subject Health Status	Mean Difference (I-J)	Std. Error	CI 95%	
					Lower Bound	Upper Bound
CS Social support seeking	VIH	Diabetes	−6.81 *	0.689	−8.43	−5.18
CS Problem-solving coping	VIH	Diabetes	−2.56 *	0.589	−3.95	−1.17
CS Positive reappraisal	VIH	Diabetes	−1.74 *	0.489	−2.89	−0.58
CS Negative auto-focused coping	VIH	Diabetes	2.45 *	0.402	1.50	3.39
CS Overt emotional expression	VIH	Diabetes	0.61	0.416	−0.37	1.59
CS Religious coping	VIH	Diabetes	−3.96 *	0.771	−5.78	−2.14
CS Avoidance coping	VIH	Diabetes	−2.10 *	0.493	−3.26	−0.93

The Dunnett C test was used. * significant differences; CS: coping strategies; Std. error: standard error; CI: confidence interval.

## Data Availability

The data that support the findings of this study are available on request from the corresponding author. The data are not publicly available due to privacy or ethical restrictions.
